# The role of place-based factors and other social determinants of health on adverse post-sepsis outcomes: a review of the literature

**DOI:** 10.3389/femer.2024.1357806

**Published:** 2024-02-29

**Authors:** Robert R. Ehrman, Adrienne N. Malik, Brian D. Haber, Seth R. Glassman, Cassidy A. Bowen, Steven J. Korzeniewski, Samantha J. Bauer, Robert L. Sherwin

**Affiliations:** 1 Department of Emergency Medicine, School of Medicine, Wayne State University, Detroit, MI, United States,; 2 University of Kansas School of Medicine, Kansas City, KS, United States,; 3 Jacobs School of Medicine and Biomedical Sciences, University at Buffalo, Buffalo, NY, United States,; 4 University of Kansas School of Medicine-Wichita, Wichita, KS, United States,; 5 Department of Family Medicine and Public Health Sciences, School of Medicine, Wayne State University, Detroit, MI, United States

**Keywords:** sepsis, social determinants of health, readmission, environmental factors, social risk factors, mortality, post-discharge outcomes

## Abstract

Sepsis remains a common and costly disease. With early recognition and guideline-based treatment, more patients are surviving to hospital discharge. Many survivors experience adverse health events in the months following discharge, while others suffer long-term physical and cognitive decline. Social, biological, and environmental factors affect all aspects of the disease process, from what pathogens one is exposed to, how/if disease develops, what avenues are available for treatment, as well as short- and long-term sequelae of survival. Disparities in sepsis care exist at all stages of a patient’s clinical course, but increased survivorship has highlighted the extent to which Social Determinants of Health (SDoH) influence post-discharge adverse events. Despite increased interest in the last decade, a nuanced understanding of causal relationships remains elusive. This is due to several factors: the narrow range of social determinants of health (SDoH) variables typically studied, the inconsistent and non-standardized methods of documenting and reporting SDoH, and the inadequate acknowledgment of how social, environmental, and biological factors interact. Lack of clear understanding of how SDoH influence post- discharge outcomes is an obstacle to development and testing of strategies to mitigate their harms. This paper reviews the literature pertaining to the effects of SDoH on post-discharge outcomes in sepsis, highlights gaps therein, and identifies areas of greatest need for improving the quality and impact of future investigations.

## Introduction

Sepsis affects millions of patients each year, with estimated associated costs in the United States (US) of $38 billion annually (1). Sepsis-associated mortality has decreased in the last two decades (2, 3), but increased survivorship brings new challenges for patients and healthcare systems. Sepsis survivors experience a high frequency of re-admission [up to 26% at 30 days (4) and 43% at 90 days (5)], with estimated costs up to $17,000 per visit, and >$3.5 billion annually in the US (6). While infection/sepsis is the most common reason for re-admission, exacerbation of underlying medical problems is also frequent, and presence of chronic conditions (e.g., heart failure, kidney disease, diabetes) is associated with greater risk for re-admission (7).

Racial disparities in sepsis incidence and outcomes have been reported, but substantially less data exist about how social variables—other than race and socioeconomic status—contribute to such disparities (8, 9). Similar inequities have been reported in terms of adverse post-discharge events, but extant data is limited in terms of the social variables that have been assessed, and investigation of the impact of environmental factors is lacking (10). The effects of complex interactions between individual patient characteristics and place-based/area-level factors (such as areas with greater social deprivation or exposure to pollution) remain unexplored. These gaps in our understanding preclude the development of strategies to reduce these harms and address the long-term morbidity and mortality of sepsis, which has been identified as one of the top priorities in sepsis research (11).

There is increasing recognition of the impact that Social Determinants of Health (SDoH) have on the provision of emergency healthcare (12). For example, health literacy and other SDoH factors have been found to affect ED recidivism (13, 14). Post-discharge readmissions are relevant to Emergency Medicine as most hospital admissions begin with an ED visit. Understanding how SDoH affect patients with sepsis—at initial visits and revisits—will help emergency physicians provide optimal care. Research focused on design and implementation of strategies lessen the deleterious effects of SDoH should involve Emergency Medicine because the ED is the initial point of contact with the healthcare system for so many patients. Given that EDs provide care for diverse populations, including vulnerable groups who experience an abundance of social stressors, such efforts are likely to provide benefit for many patients, not only those with sepsis.

The goal of this paper is to review the literature on risk factors for adverse outcomes after discharge from a sepsis admission, with a focus on place-based factors (social, environmental) and their interactions with patient-level characteristics. Within this framework, limitations of existing data, as well as barriers to collection of reliable and accurate SDoH measures will be discussed. Finally, analytic considerations for future studies in this area will also be explored. As a narrative review, literature search and decisions for inclusion/exclusion were at the discretion of the authors.

## What are social determinants of health?

Social determinants of health are the intrinsic qualities of the areas where people are born, live, work, receive their education, socialize and worship.^1^ The environmental influences inherent in diverse settings lead to differences of health outcomes, manifesting as variations in quality of life, physical function, and disease susceptibility (15–18). SDoH are stratified into five domains: economic stability, education access and quality, healthcare access and quality, neighborhood and built environment, and social and community access (15, 16). SDoH are distinct from social *risk factors* in that SDoH exert an effect on everyone, cannot be modified on an individual level, and are not inherently negative in their impact. Social risk factors occur on a personal or family unit level and can sometimes be modified at that level to improve or constrain health, although typically the term is used to refer to “specific adverse social conditions associated with poor health” (15, 19). The most influential of the social risk factors in determining health on an individual level are income, wealth, education, occupation, race, gender identity and social equality attributable to race or ethnic group (20). A brief, comparative overview of terms/definitions is provided in Table 1.

Social risk factors and SDoH are separate still from behavioral risk factors, which also occur on an individual level and include negative habits such as substance use, smoking, unhealthy eating, and lack of exercise (15). Social risk factors heavily influence and intersect with behavioral risk factors to compound health disparities. It should be noted that these are distinct, and independent of mental health risks, though they both exert obvious influence on the role of mental health and overall wellness at an individual level (15, 18). Individual-level factors also include immutable characteristics such as genetics, age and the biology of chronic disease states, which are discussed separately in this paper.

Though separate from social and behavioral risk factors, SDoH can influence both. Evidence indicates that overall, SDoH and social and behavioral risk factors may have more influence than direct medical care in determining who becomes sick or injured than previously thought (15, 17). For example, a meta-analysis that utilized data on deaths in the United States in 2000 reported that mortality attributable to low-education, racial segregation, and inadequate social support were comparable to those attributed to lung cancer, cerebrovascular disease and myocardial infarction, respectively (21).

SDoH have broad reaching, significant effects on health and medical outcomes at both individual and population levels. Far from being as simple in their influence as their aforementioned domain categories, SDoH are complicated and multidimensional. SDoH exert influence on health from micro to macro levels, and are complexly intertwined in their temporal relationships with each other and with social and behavioral risk factors (15–17). SDoH begin impacting populations at the macro level of government and institutional policy and through their effect on laws and distribution of wealth and resources. They exert further downstream effects on a more individual level by influencing jobs, community access to food, money and necessities of daily living (15). Additionally, they are inexorably connected and influenced by each other on multiple levels. For example, physical environment and social environment can influence one another such that poor interpersonal relationships in the presence of deleterious social networks may increase crime in a neighborhood, which directly affects area businesses and infrastructure leading to poor physical environment. This, in turn, may negatively influence interpersonal relationships, thus creating a negative feedback loop effecting multiple aspects of SDoH of an entire community. The additive effect of SDoH can be exemplified by the findings from a large, retrospective study examining the association between income and life expectancy in the United States, which found a compounding effect from SDoH that led to the poorest 1% of the population having an associated 10–15 year shorter life expectancy than the richest individuals in the cohort (22).

Intersectionality is an additional concept integral to understanding the influence of SDoH on overall health and barriers to medical care. Butkus et al. (18) describe intersectionality as a theoretical framework positing that multiple social categories (e.g., race, gender, sexual orientation, socioeconomic status) intersect at the level of individual experience to reflect multiple intertwined systems of higher-level oppression and privilege, borne out at the societal structural level (e.g., racism, gender discrimination). Intersectionality leaves some individuals exceptionally vulnerable to negative influence from multiple facets of SDoH if they are simultaneously members of more than one social category. Assessing disease states, health disparities, and barriers to care through the lens of intersectionality helps avoid inappropriately attributing negative outcomes to individual level factors and instead examines the causality from a multifactorial and systemic perspective (18, 23).

## What are adverse post discharge outcomes?

Re-hospitalization is amongst the most common post-discharge events for sepsis survivors. A meta-analysis from 2020 reported mean re-hospitalization at 30-days of 21.4% (95% CI 17.6–25.45%) and 38.1% (95% CI 34.3–42.0%) at 90-days (24). Infection or a repeat episode of sepsis is the most common reason for readmission, accounting for up to 60% in some cohorts (4). Exacerbation of chronic medical conditions (e.g., heart failure, COPD, chronic kidney disease) occurs with individual frequencies of 1%−4%, and nearly 20% when combined (25). Some admissions for “ambulatory care sensitive conditions” (COPD, heart failure, etc.) are thought to be preventable in the general population, but whether this is true after surviving sepsis requires further study (25). Surviving a sepsis event is associated with overall increased utilization of inpatient healthcare facilities (hospitals, skilled nursing facilities, and long-term acute care centers) up to 2 years after index hospitalization (26). Prescott et al. reported severe sepsis survivors spent more days (16 vs. 7, *p* < 0.001), and a greater proportion of days (9.6 vs. 1.9 %, *p* < 0.001), in a facility in the year after hospitalization, compared to the year before. While this was similar to survivors of non-sepsis-related hospitalizations, sepsis survivors had substantially greater post-discharge mortality (44.2 vs. 31.2%) and fewer days at home (difference-in-differences, −38.6 days, *p* < 0.001) (26).

Other long-term sequela of sepsis are reported in survivors. In a cohort of 516 sepsis survivors with a mean age of 76.9 years, there was a 10.6% increase in moderate to severe cognitive impairment (27). There was also an increase in new functional limitations both for those with prior limitations (mean 1.5 new limitations) and those without (mean 1.57) (27). Patients who survive sepsis-related Acute Respiratory Distress Syndrome, despite recovering to nearly normal pulmonary function, experience long-term reduction in exercise capacity (only 76% of predicted distance on the 6-min walk test), and physical quality of life (28). A meta-analysis of 38 studies found that one-third of survivors from ICU admission experience depressive symptoms up to 12-months after discharge (29). Development of anxiety (32%) and post-traumatic stress disorder (44%) also occurs (30).

Multiple factors contribute to these adverse outcomes, including new or worsening organ function, resultant increased need for care, and immunomodulation (30). Some patients develop Persistent Inflammation, Immunosuppression, Catabolism Syndrome (PICS), although development of PICS is not limited to patients with sepsis. Contributors to PICS are incredibly diverse, including genetics (31, 32), lipids (33), and diet (34). Sepsis patients with hyperinflammation and immune suppression, compared to those without this phenotype, have greater all-cause and cardiovascular-related mortality and readmission at 6-months [hazard ratio (HR) 1.53 and 5.07, respectively], and greater 1-year mortality (OR 8.26) (35). Social and environmental factors are likely to play a role in development of immune dysfunction but remain understudied.

## Place-based factors: an overview

“Place” refers to factors located within a particular geographic area, including schools, housing, recreational facilities, and retail stores. Environmental factors, such as air quality, also contribute important place-based characteristics. Place is a critical factor to consider from a public health standpoint (36) as characteristics of place are inextricably tied to health outcomes (8, 9, 37, 38), yet health-promoting features are inequitably distributed (39).

Healthcare-related factors, such as living in a medically underserved area [defined using (1), the ratio of primary care physicians per 1,000 people, (2) the infant mortality rate, (3) the proportion of the population with income below the poverty level, and (4) the percentage of the population ≥65 years of age] have been associated with increased adverse health outcomes for innumerable conditions (18, 39–42). Many stand-alone (e.g., race, income, education) and combined metrics of socioeconomic stress [e.g., Social Vulnerability Index (SVI), Neighborhood Deprivation Index (NDI), and Area Deprivation Index (ADI)] across variably-sized geographic areas (zip code, county, census tract) have found reduced healthcare access and worse health outcomes in areas with greater socioeconomic disadvantage (38, 43–45).

Lifestyle factors, such as diet and exercise, have been linked to health outcomes across myriad conditions. While historically, diet and exercise have been referred to as “lifestyle choices,” current evidence shows that place-based features—by determining what resources are available within a geographic area—may be substantial drivers of “choice” (46, 47). The landscape of food availability varies widely, with low-income areas often having greater density of fast-food restaurants and convenience stores, with fewer full-service supermarkets, when compared to high-income areas (47–49). The latter offer more healthful options (e.g., fresh fruits and vegetables) while the former two largely offer highly-processed foods that tend to be more calorie dense, and high in sodium, fat, and sugar. A plausible link between living in a food desert (defined as areas with limited access to affordable healthy food) and development of chronic conditions such as diabetes, hypertension, and coronary artery disease exists (50). However, food deserts often occur in low-income areas where this and other socioeconomic stressors may confound the relationship between food availability and health outcomes (51–53).

Physical activity has a strong relationship with health status, and there are established benefits for primary and secondary prevention across many diseases (54). In general, patients with the lowest levels of physical activity tend to have greater all-cause mortality (55–57) and disease-specific adverse events (58, 59) than more active counterparts. Place-based factors influence physical activity, as spaces are needed for such endeavors (parks, trails, gyms, etc.) and they need to be safe and accessible (both in terms of distance and affordability). Poor access to such spaces can act as a barrier to being physically active, and thus increase risk of disease. Distribution of spaces for exercise is unequal, as low-income areas often have fewer parks. Crime or violence maybe deter people from being active in their own place of residence or traveling to/from recreation areas. A variety of socioeconomic status (SES) variables have been linked with reduced levels of physical activity (60), but as with diet, understanding the influence of place-based factors—alone or in combination with other SES measures—remains challenging and understudied.

## Place-based factors: outcomes and evidence

A variety of place-based factors have been shown to be associated with greater incidence of sepsis and increased adverse outcomes. Rate of hospitalization for sepsis was found to be greater in medically underserved areas (8.6 vs. 6.8/1,000 people, *p* < 0.1); inpatient mortality rate was also increased (15.5 vs. 11.9/10,000, *p* < 0.1) (8). Areas with lower overall educational attainment, greater income inequality, and rural location have increased sepsis-related mortality (9). Greater mortality is also seen in locations with fewer physicians per capita (9), a potential indicator of limited access to healthcare. Another potential contributor is between-hospital differences in quality of care. For example, a study conducted in New York City found that hospitals serving predominately minority populations were less likely to provide guideline-adherent sepsis care (61). Another study reported delays in antibiotic administration for Black patients with septic shock, compared to White patients (62). Environmental factors also influence sepsis-related outcomes. In a cohort of 53 million Medicare beneficiaries, a mean increase of 10 μg/m^3^ in small particulate matter (PM_2.5_) exposure over 1-year increased incident sepsis mortality risk by 9% (63).

Similar place-based characteristics are likely to influence post-discharge events, although less evidence exists in this domain. A study from an urban academic medical center found that mean ADI (higher scores indicate greater disadvantage) was greater in patients re-hospitalized at 30-days compared to those who were not (62.5 vs. 51.8, *p* < 0.001) (64). In contrast, a study of >1.3 million Medicare beneficiaries found no relationship between ADI and 30-day readmission (65). Mortality—which is a competing risk for readmission—was greater in the most deprived (ADI = 100), vs. least deprived (ADI =1) areas (OR 1.35, CI 1.29–1.42), which could distort the ADI-readmission relationship. Overall, however, one cannot conclude that the latter study is “correct” simply due to larger sample size. Differences in model assumptions and covariates, included patients (all patients with sepsis vs. only Medicare enrollees), or well-documented potential for errors in large administrative data sets (see “Limitations” section) could explain conflicting results. In addition, area-level factors may demonstrate heterogeneity of treatment effect (HTE): the effect of ADI on 30-day readmissions may vary by area. In the single center study in Baltimore, ADI has a detectable association with readmission whereas in the study using place-aggregated data, positive associations in certain locations may be diluted by negative or null associations in others, resulting in the finding of no overall association. The implications of HTE in studies of critically ill patients is a well-recognized phenomenon (66).

## Sepsis and social risk factors: outcomes and evidence for individual-level, non-biologic factors

Health disparities related to individual social risk factors such as race, SES and gender are found throughout healthcare, but with regard to sepsis outcomes and hospital readmissions, minority groups are noted to have a greater adjusted incidence of hospitalization, disease-related complications, and divergence from standard care when compared to white counterparts (67–69). Variations across racial and ethnic groups have been reported, and despite the association between minority identity and living in an impoverished area, studies have found that Black individuals had a greater incidence of severe sepsis compared to other racial identity groups, while Hispanic individuals were found to have a lower incidence of severe sepsis compared to white counterparts (70, 71). In a large retrospective study by Chang et al. (4), patients identifying as Black or Native American had associated greater odds of 30-day readmission following sepsis hospitalization (OR 1.29 and 2.39, respectively). Another study evaluating factors associated with readmission following index sepsis hospitalization noted that race along with age, SES and comorbid disease burden were all associated with readmission (7). Attributing these differences in sepsis outcomes and severity solely to racial identity incompletely represents the causality of the findings, however, and likely fails to capture the true gamut of influences underlying these data as minority race is often a proxy identifier for poverty, chronic disease states and inadequate, healthcare access (4, 72).

Likewise, sepsis data related to income level show disparities between individuals of higher income, compared to those of lower income, as well as homelessness (a proxy for low or lack of income) (4, 72). In the United States, SES is a strong predictor of health outcomes as individuals with low SES tend to lack health insurance or easy access to primary care, and are more likely to exhibit behaviors that contribute to overall poor health (72). Additionally, individuals lacking health insurance are more likely to present to a hospital for sepsis care at a more advanced state of disease and thus, lack of insurance is independently associated with greater sepsis mortality (73, 74). A large retrospective study of ICU patients in 2019 found that homeless ICU patients with sepsis did not experience a greater degree of mortality or longer ICU LOS compared to their non-homeless counterparts but did experience significantly longer overall hospitalization (75). This finding was despite the greater burden of other social risk factors in the homeless population such as substance abuse, mental illness and liver disease. The authors propose that this LOS difference was due to difficulties arranging post discharge follow up and appropriate step-down locations for further outpatient recovery (75). Sub-optimal post-discharge care in this group may increase risk for re-admission, a hypothesis supported by a statewide retrospective study of sepsis readmissions that found that lower-income level was associated with increased odds of readmission at 30 days following an inpatient stay (4). While SES strongly predicts individual health, truly quantifying the impact of SES on sepsis outcomes is difficult, as income level is often a surrogate for neighborhood, race and education (75).

Data pertaining to sepsis-related mortality based on gender are limited and conflicting. Incident cases are greater in males, but studies generally do not show a significant outcome difference between gender groups, although the overall quality of evidence is low (76, 77). In terms of re-admission, one retrospective study found male gender was associated with greater odds for 30-day readmission following sepsis hospitalization (4). However, current data evaluating the influence of gender on sepsis outcomes and readmission is fraught with multiple confounders, including the lack of a specific definition for gender in most studies, general non-inclusion of pregnant patients (despite sepsis causing 11% of maternal deaths) and a lack of adjustment for baseline comorbidities (7, 72, 76). Most data on this subject was obtained evaluating gender through a binary, social lens, or without explicit definition of what is meant by male or female gender (7, 18, 23). According to a scoping review by Miani et al. (23) of gender as a SDoH, “… In this context, gender should be understood as an individual’s socially ascribed attributes, roles, responsibilities, and expectations in a given society based on their gender expression and how others perceive it, in contrast to sex being about the biological, physiological, genetic and hormonal bodily characteristics of a person.” With the increasingly broad definitions and characterizations of gender identity, the knowledge that some members of the LGBTQIA population tend to delay seeking healthcare or avoid it altogether, and are less likely to possess health insurance, it stands to reason that sepsis outcomes are likely to be influenced by specific gender identities (18, 76). Future investigations assessing the influence of gender on sepsis outcomes should utilize more precise dimensions and definitions of gender identity and evaluate their data through a lens of intersectionality.

## Biologic factors: outcomes and evidence

In this context, we define “biologic factors” as conditions or states that occur at the level-of the individual that are the result of deranged physiology and/or departure from normal function of an organ or the body as a whole. Overall, worse pre-septic health status is associated with greater likelihood of adverse long-term outcomes (25). An enormous volume of literature exists in this area, and the following paragraphs represent a brief overview of the most proximate person-level associations.

A large cohort study from hospitals across the United States found that malignancy, diabetes, and chronic diseases of the heart, lungs, liver, and kidneys were associated with increased OR (range 1.11–1.34) of 30-day readmission (78). Similar risk factors and effect sizes are reported in other studies (6). Younger patients tend to have increased risk of re-admission (4, 78), but some of this effect may be related to poor functional status in older individuals and patients who transition to palliation rather repeated hospitalizations (7, 25). Characteristics of index sepsis hospitalization associated with increased risk of readmission include ICU admission, longer length of stay, and discharge to a nursing home or other healthcare facility (7, 24).

A limitation of all these studies is that while biologic factors may be multifactorial in etiology (genetics, obesity, and tobacco use all contribute to hypertension), studies linking biologic factors to sepsis readmissions tend to be cause-agnostic. This is at least in part because determining how much each etiologic agent contributes to blood pressure elevation (or other physiologic derangement) is difficult or impossible. Each contributing factor likely has pleiotropic health-effects, which adds further complexity to these relationships. Analytic strategies that attempt to mitigate some of this confounding do exist (see “Analytic considerations” section), but they are unlikely to completely solve the problem. Thus, a pragmatic approach is to consider biologic factors as potentially modifiable risks for adverse post-discharge events while acknowledging that sufficient heterogeneity exists such that the effect of a given biologic factor may vary across populations or locations.

## The intersection of biologic, social, and environmental factors

For any individual, overall health is influenced by, and dependent upon, multiple factors. Some biologic factors are immutable, such as age and genetics, while others may vary widely both within- and between-individuals (weight, blood pressure, etc.). These person-level biologic factors interact with individual (e.g., education, income, health literacy) and area-level SDoH variables (e.g., neighborhood or census tract SVI). For example, the effect of living in an area with high SVI may be different for an obese male with a strong family history of coronary artery disease than for a non-obese person with a less at-risk genetic profile. Environmental factors, such as exposure to fine particulate matter (PM_2.5_) may increase heart failure incidence and mortality (79), but there is likely to be effect modification by both biologic and social factors.

These relationships may be further confounded by the fact that some factors that are considered “social,” including race, may have biologic and non-biologic components. In the United States, there is increased incidence of hypertension in African Americans, with some of this risk attributable to genetic predisposition (80). Undoubtedly, however, race is also a proxy (although imperfect) for structural health inequities, such as racism, poverty, and exposure to environmental pollutants, all of which have deleterious effects on health. Furthermore, persistent segregation tends to influence the physical and social landscape of neighborhoods through perpetuation of historically established concentration of poverty and limited access to education and employment opportunities (39). These same factors also lead to inequities in access to healthcare, further eroding individual and community health (81). Note that we do not, in any way, promulgate the notion of a biologic basis for initiation or perpetuation of social inequities. Rather, any social construct, regardless of how or why it was created, will, by definition, have a biologic component because it pertains to humans.

The intersectionality of biologic, social, and environmental factors plays a key role in the development of infections, progression to sepsis, recovery, and post-discharge outcomes. Socially disadvantaged groups experience greater burden of a variety of infectious diseases (82–84), with individual-level factors (e.g., diabetes, HIV, tobacco use) increasing risk for adverse outcomes. Focus on the individual level is insufficient, however, as it ignores what puts people at risk for risks. *Fundamental Cause Theory* posits that social and environmental factors function as upstream causes of disease as they influence so many aspects of life that affect health—food, housing, education, personal safety, and access to healthcare (85). Structural racism, for example, influences where a person can live, work, and play, as well as what they eat; it is also tied to other factors, such as economic disparity, environmental pollution, and reduced access to healthcare and education. The multitudinous pathways, however, connecting structural inequities to adverse health outcomes remain incompletely described.

Noppert et al. (86) developed a conceptual framework as a starting point in the explanation of the complex relationships between social and environmental factors and infectious disease disparities. They hypothesize that this occurs via two related pathways: (1) increased pathogen exposure (via the household, the neighborhood, and the work environment) and (2) greater susceptibility to infection (via stress, underlying heath condition, and immune suppression) (86). They posit that while exposure and susceptibility occur at the individual level, they are inextricably linked to the exposome, which itself is heavily influenced by structural factors, such as economic disadvantage and racism.

Many biologic factors that predispose to infection (e.g., diabetes, HIV, lung disease) are themselves influenced by the exposome. These same factors (and thus, the exposome) affect post-discharge outcomes in many ways, including greater need for follow-up care, given their medical complexity, as well as potentially constrained access to care. Strategies to improve post-discharge outcomes for patients with sepsis must, therefore, include attention to proximal causes of disease. Such approaches will have the added benefit of potentially reducing the risk of developing infection (by reduced exposure and susceptibility) and reduced incidence of conditions that predispose to adverse outcomes.

## Limitations and difficulties in collecting SDoH

Recognition of the impact of SDoH on post-discharge sepsis outcomes is rising. A recent scoping review found the number papers considering SDoH factors as contributors to post-sepsis mortality and readmission has increased in the last two decades (10). However, of 103 articles selected for full review, only 28 (27%) reported any SDoH factors, suggesting that this this remains a relatively understudied topic. The most often studied SDoH factor was “race/ethnicity” (*n* = 21 studies, 75%); income/wealth (*n* = 8, 29%), and insurance payor type (*n* = 10, 36%) were the next most common. A paucity of the included papers considered environmental characteristics (*n* = 6/21% for both urban vs. rural residence and neighborhood socioeconomic status). None of the studies specifically discussed structural racism or individual physical characteristics of the environment, nor did any study consider social-biologic-environmental interactions.

There are many challenges in the collection and reporting of SDoH data. These variables are often obtained from the EHR or other administrative datasets, despite evidence that these sources are prone to errors or missingness (87). Some SDoH factors change over time (education, income, etc.), but whether these changes are accurately incorporated into existing data sources is unclear (88). Obtaining SDoH data directly from patients, while considered the gold standard, is challenging, owing to multiple factors: time constraints on persons obtaining information, lack of standard definitions and reporting methods, and reluctance by patients to provide information that they may feel has pejorative connotations. None of the studies in the review by Hilton et al. validated their SDoH data, and data missingness was not reported in 71% of included studies (10). Errors and omissions in source datasets are thus likely to persist, potentially biasing results of analyses thereby distorting the true relationship between SDoH variables and outcomes. Furthermore, extant datasets include only a small proportion of SDoH factors likely to influence health outcomes (89), rendering existing studies incomplete.

Consideration of “race” as an SDoH variable deserves special attention. Race is often used as a proxy for racism, but this is problematic, for multiple reasons. First, errors in recording race and ethnicity in administrative datasets have been recognized for decades (90), and while accuracy has improved, misclassification persists (91). More importantly, racism is not a single entity but rather a manifestation of unequal opportunities across multiple domains—access to housing, employment, education, healthcare, etc. Structural inequities at any of these levels are not only unjust, but grouping the varied domains of racism into a single indicator variable (“race”) lacks scientific rigor. To posit that all components and manifestations of structural racism act uniformly at the individual, neighborhood, or population level, at discrete timepoints or over the life course, is entirely implausible. Use of “race” will thus capture some of the effects of racism, but without sufficient detail to develop targeted interventions based on root causes.

## Analytic considerations

While a complete discussion of the varied statistical methods applicable to this area of research is beyond the scope of this paper, we wish to highlight two concepts germane to the study of social, biologic, and environmental interactions in the setting of sepsis and adverse post-discharge outcomes.

The first is the concept of *time-varying risk of infection*. There is established seasonal variation in spread of some infectious diseases (e.g., influenza) owing to pathogen, host, and environmental factors (92, 93). However, relative risk of infection for a given pathogen in one population, compared to another, have historically been approached as fixed over time (94). This tacit assumption is the basis for the commonly used Cox Proportional Hazards model. While a biologic factor may increase risk for certain types of infections, such as chronic lung disease predisposing to respiratory pathogens, considering this relationship as time-invariant ignores potential influence of social and environmental factors. This is particularly important in the immediate post-discharge phase of a sepsis admission, when patients are most likely to suffer a setback leading to readmission.

At the patient level, time-varying risks may influence post-sepsis outcomes in myriad ways, such as increased susceptibility to new infections or re-infection, which itself may vary based on the original source/type of infection that led to the sepsis episode. Re-infection (and thus potential for re-admission) risk may be moderated by particulate matter exposure or residing in a socially disadvantaged area, but these factors may operate on different time scales, and with different magnitudes based on patient-level characteristics. From a population level, understanding how infectious disease risk varies over time can lend insight into core epidemiologic questions, including which populations are at risk for contracting or transmitting infections (94). Characterizing these time-varying risks is a key first step toward designing mitigation strategies at both the individual and area-level.

The second analytic concept we wish to highlight is *time-oriented analysis of risks* (95–97). When researchers seek knowledge about what factors are associated with a particular outcome, a common approach is to build a regression model with putative risk factors entered as covariates. The analyst chooses criteria for retention of covariates in the model, such as statistical significance or the effect of adding a covariate to model fit (e.g., Bayesian Information Criteria). Such approaches ignore the fact that certain exposures (risk factors) occur in an ordered fashion. For example, age and biological sex operate/occur ahead of downstream phenomena, such as smoking status or body mass index. At the community level, air pollution or residing in a high SVI census tract precedes person-level evidence of need for re-admission on presentation to the Emergency Department. Failure to consider time-order can lead to elimination of early exposures as their effect (s) are “overpowered” by those that come later. A time-oriented analysis of risks approach instead carefully selects parameters into small clusters of two-to-three parameters that are not multi-colinear (known as “epochs”), and then examines magnitudes of association sequentially with respect to time-order. Factors found to be significantly associated in each epoch are retained in all subsequent models, regardless of changes in statistical association for the retained variable(s). This strategy helps ensure that variables whose influences operate early are not supplanted by those measured later, which is of particular import when the goal is the earliest possible prediction of adverse outcomes.

## Discussion

Sepsis remains a common and costly disease. As survival from the index event improves, there is increased need to understand the long-term sequelae and factors that influence outcomes. As highlighted by Fundamental Cause Theory, SDoH are likely key contributors to adverse post-discharge events.

Successful interventions have been described, such as use of a nurse lead post-discharge transition service, which reduced the odds of death or readmission compared to usual care at both 30-days (OR 0.80, CI 0.64–0.98) and 12-months OR 0.70 CI 0.50–0.98 (98, 99). While encouraging overall, the only SDoH variable included in these reports was “Race” (Black vs. White vs. Other), so further investigation from an SDoH standpoint is needed. Despite the increase in studies in this area in recent years, the overall body of literature has several key limitations, which act as obstacles to design and implementation of targeted interventions.

**FIGURE 1:**
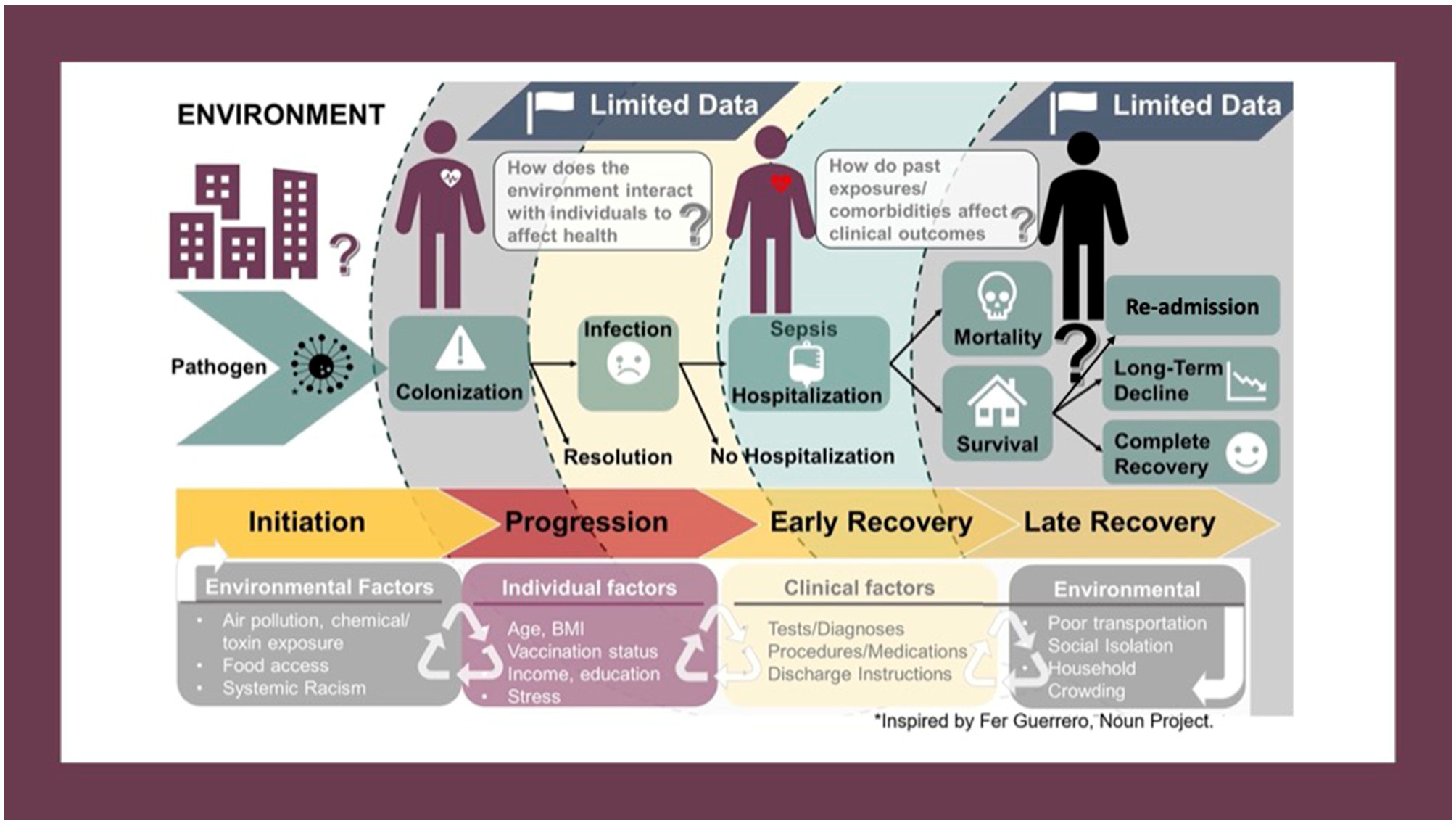
Conceptual framework depicting the potential pathways through which social, biological, and environmental factors can affect the infection-recovery pathway. The process is cyclical, not linear, as the same factors that affect development of sepsis also affect disease progression, recovery, and post-discharge outcomes. Interaction between SDoH factors also occurs, as does effect-modification and/or mediation.

Most importantly, methods for collection and reporting SDoH factors lack the scientific rigor given to other health data. Standardized definitions, collected directly from patients, with regular updates, are needed to ensure accuracy and reliability. As illustrated by our conceptual framework (Figure 1), the relationship between SDoH and health outcomes is (1) cyclical, and (2), extraordinarily complex. The process is cyclical (not linear) because each exposure is influenced by prior exposures and events while also influencing subsequent events/exposures. The same factors that affect risk of developing sepsis also affect progression, recovery, and long-term outcomes, including re-admission. There is additional complexity owing to the longitudinal nature and time-varying effects of the exposome, difficulty quantifying effects of multifaceted social phenomena (e.g., racism), and effect modification and/or mediation of varied SDoH variables on health outcomes. Interactions amongst SDoH factors—particularly between place-based and individual factors—remain largely unexplored.

Our conceptual framework was developed using ideas from Syndemic Theory. The syndemic health model focuses on the synergistic relationship between people, their health, and their environment, rather than considering diseases as stand-alone entities that act independently across space and time (100). Approaching sepsis through a syndemic framework has been suggested as a way to improve disparities in sepsis-related outcomes (101). This is, however, a departure from the traditional paradigm that a given disease requires the same treatment, regardless of the patient and their circumstances. The ideological shift is another challenge to the study of how SDoH impact post-discharge outcomes in sepsis. Success will require collaboration across multiple medical specialties (Emergency Medicine, Critical Care/Inpatient Medicine, and outpatient clinics) and other stakeholders who provide healthcare-related services (e.g., social workers, physical therapists, skilled nursing facilities).

Existing literature covers only a limited number of variables, and further studies addressing the full spectrum of SDoH are needed to fill knowledge gaps. Main results from selected studies included in this review that we wish to highlight are available in Table 2. Careful interpretation of current and future studies is warranted, as findings of studies from one location may not transport/generalize to another. This is not because the findings are untrue, but rather, because exposomes are highly variable. Local characteristics (pathogens, social conditions, etc.) can act together to create unique exposomes, such that associations from one geographic location simply do not exist in others. A corollary to this is that aggregated location data may bias toward null associations as local effects are diluted in pooled data sets.

There is no debate that SDoH factors have a strong influence on health outcomes. Consideration of these issues may seem outside the scope of emergency care where the focus is on the acute, presenting condition, rather than the factors that influence development and outcomes of said condition. EM practitioners often consider problems like poverty, structural racism, or pollution as falling within the purview of Public Health, as opposed to EM (102). Surveys have demonstrated that while Emergency Physicians feel public health issues are important, directly addressing them during clinical shifts is challenging (103, 104).

In 2023, the Evaluation and Management coding guidelines from the American Medical Association added SDoH as a documentation component that contributes to medical complexity, an indication of the increasing recognition of the impact SDoH have on health outcomes.^2^ As these codes are used for billing EM charts, emergency care providers are perhaps more aware than ever of the importance of SDoH. The challenge moving forward will be to ensure that SDoH are truly incorporated into medical decision making for initial and repeat sepsis visits (and others) rather than simply being added to charts solely for billing purposes. A critical step toward achieving this goal is accurate collection and thoughtful analyses of these data in order to provide a detailed understanding of exactly how SDoH affect ED patients. Quantitative data are needed to design and test interventions aimed at reducing deleterious effects. Quantification has the added benefit of taking an abstract concept (e.g., “living in poverty is harmful”) and making it more concrete (e.g., living in a high SVI neighborhood conveys a five-fold increased risk of 90-day mortality after discharge from a sepsis admission). The latter is easier for an Emergency Physician to act upon when seeing a recently discharged patient during a busy ED shift.

Social, biological, and environmental factors play an integral role in health maintenance and exposure to, development of, and recovery from disease, including sepsis. Scientific investigations in this area by acute care specialties are increasing, but the volume of literature is miniscule in comparison to studies of direct medical interventions. To be sure, the latter have improved sepsis-related mortality in the last two decades (2, 3), but disparities in short- and long-term outcomes remain. As detailed in this review, studying SDoH, quantifying direction and magnitude of effects, and elucidating causal pathways (or even associations) is difficult. Identifying and overcoming these challenges and pursuit of scientifically rigorous research in this field is necessary so that high-quality, equitable care can be provided to all patients.

## Figures and Tables

**TABLE 1:** Definitions, examples, and outcome associations for key terms used in the manuscript.

Term	Definition	Key points	Examples	Adverse post-discharge outcome associations
Social determinants of heath (SDoH)	*Intrinsic qualities of areas* where people are born, live, work, etc. that affect health of all individuals in a particular area	Umbrella term for non-individual, non-biologic factors Not inherently negative Not modifiable at the *individual* level	Literacy rate, Social Vulnerability Index, healthcare and school access, crime rate	Greater degree of vulnerability and disadvantage associated with readmission, cognitive/physical decline and 1-year mortality Extensive confounding exists owing to lack of consistent definitions and robust data collection methods
Social risk factors	*Individual level*, non-biological (i.e., social) characteristics that negatively affect health at the person-level	Distinct from SDoH but often used interchangeably May be modifiable	Income/wealth, level of education, occupation, social equality attributable to gender identity, ethnicity, or race	
Behavioral risk factors	Person-level activities that adversely affect health	May be tied to or influenced by SDoH and/or social risk factors	Smoking, substance use, sedentary lifestyle, non-salubrious food consumption	Worse baseline health associated with ↑30-day readmission and 1-year mortality
Place-based factors	Characteristics of the natural and/or built environment that affect heath directly or indirectly	Some overlap with SDoH Explicitly includes *physical* aspects of an area while SDoH does not	Pollution, access to parks/areas for exercise/recreation, transportation infrastructure, full-service food stores	Sparse data for physical factors limits conclusions that can be drawn
Biologic factors	Conditions resulting from abnormal physiology or organ dysfunction	May be acquired or inherited May be modifiable	Diabetes, heart failure, obesity, etc.	Greater co-morbidity burden associated with ↑30-day readmission and 1-year mortality

**TABLE 2:** Description and main results from selected studies on adverse post-discharge outcomes in patients with sepsis.

Study	Study type	Data source	Objectives	Study size	Main results
Chang et al. (4)	Multi-center retrospective cohort	Healthcare cost and utilization project state inpatient database (California), large administrative state database	Identify risk factors for 30-day readmission	240,198	Younger age (OR 1.34, CI 1.29–1.39), lower income (OR 1.13, CI 1.10–1.16), Black vs. White (OR 1.29, CI 1.24–1.33), female vs. male (OR 0.87, CI 0.86–0.89)
Gadre et al. (6)	Multi-center retrospective cohort	Healthcare cost and utilization project’s national readmission data, large administrative database	Identify predictors associated with increased 30-day readmission	157,235	Diabetes (OR 1.07, 95% CI, 1.06–1.08; *p* < 0.001), CKD (OR 1.12, 95% CI; 1.10–1.14, *p* < 0.001), HF (OR 1.16, 95% CI, 1.14–1.18; *p* < 0.001), discharge to short-/longer-term facility (OR 1.13, 95% CI, 1.11–1.14; *p* < 0.001)
Galiatsatos et al. (64)	Single-center retrospective cohort	Health database of an urban, academic medical institution	Evaluate relationships between ADI and 30-day readmission post-sepsis hospitalization	531	Mean ADI was greater for readmitted (62.5 ± 27.4) vs. not (51.8 ± 22.2), *p* < 0.001. Increasing ADI associated with readmission (beta 0.03, *p* < 0.001)
Goodwin et al. (8)	Multi-center retrospective cohort	Healthcare cost and utilization state inpatient databases (California, Florida, New York), large administrative state databases	Determine the frequency, mortality, cost, and risk factors associated with readmission after severe sepsis hospitalization	43,452	At 30 days: 26% readmitted, 4% died; at 180 days: 48% of readmitted, 8% died. Greater odds of 30-day readmission: malignancy (OR 1.34; CI, 1.24–1.45), CKD (OR 1.24; CI, 1.18–1.31), HF (OR 1.14; CI, 1.08–1.19), lung disease (OR 1.12; CI, 1.06–1.18), and diabetes (OR 1.12; CI, 1.07–1.17). Mean cost of each readmission $25,505 (SD$38,765)
Herridge et al. (28)	Multi-center prospective, longitudinal cohort	Four academic medical–surgical ICUs in Toronto, patients individually consented and enrolled	To describe the extent of physical, mental, and quality-of-life impairments after ARDS	109	At 5 years, 6-min walk test was 76% of age/sex-matched controls. 6-min walk test correlated to physical-component of the 36-Item health survey score from 3 months to 5 years (*p* < 0.01). Reduced physical quality of life scores noted 5 years after illness
Iwashyna et al. (27)	Multi-center prospective cohort	Health and Retirement Study interviews & surveys (ongoing national cohort of 27,000+ patients, ≥age 50, many with linked Medicare claims)	To describe the change in cognitive impairment and physical functioning in severe sepsis survivors	516	Increased moderate to severe cognitive impairment in sepsis survivors (OR 3.33, CI; 1.53–7.25) vs. non-sepsis patients. New functional limitations after sepsis: no prior limits, mean 1.57 new limitations (CI: 0.99, 2.15); prior mild/moderate limitations, mean 1.50 new limitations (CI: 0.87, 2.12)
Lusk et al. (65)	Retrospective cohort	United States medicare beneficiaries	To determine association between ADI and 30-day mortality and readmission in sepsis or critical illness	Mortality analysis: 1,526,405. Readmission analysis: 1,354,548	30-day mortality: greater in most-deprived neighborhoods, for patients with severe sepsis (OR 1.35, CI 1.29–1.42) or with prolonged MV with or without sepsis (OR 1.42, CI 1.31, 1.54). ADI not associated with 30-day readmission for patients with severe sepsis
Prescott et al. (26)	Observational cohort	Health and retirement study interviews & surveys (ongoing national cohort of 27,000+ patients, ≥age 50, many with linked Medicare claims)	To measure and compare pre- and post-sepsis inpatient hospital use of severe sepsis survivors, as well as compare use to that of non-sepsis severe disease survivors	1,083 severe sepsis, 1,083 non-sepsis	For survivors, more days hospitalized [median 16 (IQR 3–45) vs. 7 (0–29); *p* < 0.001] or a greater proportion of their days alive spent in a facility [median 9.6% (IQR 1.4–33.8%) vs. 1.9% (0.0–7.9%); *p* < 0.001] 1-year post-sepsis vs. 1-year prior. Sepsis survivors vs. non-sepsis survivors: greater 1-year post-discharge mortality (44.2%, CI, 41.3–47.2) vs. 31.4% CI, 28.6–34.2%); fewer at-home days (difference-in-differences, −38.6 days, *p* < 0.001); increased proportion of days spent in a facility (difference-in-differences, 5.4% (*p* < 0.001)
Prescott (5)	Retrospective cohort	Health and retirement study interviews & surveys (ongoing national cohort of 27,000+ patients ≥age 50, many with linked Medicare claims)	Identify most common readmission diagnoses after severe sepsis	2,617	42.6% re-hospitalized by 90 days: 22.2% were ambulatory care sensitive conditions (CHF, PNA, COPD, and UTI), and 11.9% were for infection
Prescott and Angus (30)	Structured review	PubMed (via search terms and synonyms for “sepsis,” “survivors”)	To provide guidance on posthospital care or recovery	N/A	1/6 of survivors have persistent impairment: new functional limits, cognitive decline, new/worsening mental health concerns (anxiety 32%, depression 29%, PTSD 44%)
Rabiee et al. (29)	Systemic review and meta-analysis	Five electronic databases	To describe depression symptoms in ICU survivors	38 studies (9 RCTs, 24 cohort, 4 cross-sectional, 1 case-series)	1/3 of ICU survivors experience clinically important depressive symptoms. Symptoms persisted through the 12-month follow-up
Shankar-Hari et al. (24)	Systematic review and meta-analysis	4 electronic databases	To assess incidence, risk factors, and reasons for re-admission after sepsis	56 studies (36 papers, 20 abstracts), all observational	Mean 7, 30, and 365-day readmissions were 9.3 (CI 8.3–10.3%), 21.4 (CI 17.6–25.4%), and 39.0 (CI 22.0–59.4%). Infection most common readmit diagnosis. Increasing age, illness severity, comorbidities, and male sex associated with increased risk
Yende et al. (35)	Multi-center prospective cohort	12 US hospitals, patients individually consented and enrolled	Identify immune-related sequelae and association with adverse long-term outcomes	483	Hyperinflammatory-immunosuppressed phenotype: ↑1-year mortality (OR 8.26, CI, 3.45–21.69; *p* < 0.001), ↑6-month all-cause readmission or mortality (HR 1.53, CI 1.10–2.13; *p* = 0.01)

OR, odds ratio; CI, 95% confidence interval; SD, standard deviation; ICU, intensive care unit; CHF, congestive heart failure; ADI, Area Deprivation Index; ARDS, acute respiratory distress syndrome; IQR, interquartile range; ARF, acute renal failure; COPD, chronic obstructive pulmonary disease; UTI, urinary tract infection; RCT, randomized controlled trial; AMI, acute myocardial infarction; PNA, pneumonia; US, United States; HR, Hazard ratio; MV, mechanical ventilation.

## Footnotes

1 https://health.gov/healthypeople/priority-areas/social-determinants-health (accessed January 12, 2023).

2 https://www.ama-assn.org/system/files/2023-e-m-descriptors-guidelines.pdf (accessed October 12, 2023).

## References

[1] Hollenbeak CS, Henning DJ, Geeting GK, Ledeboer NA, Faruqi IA, Pierce CG, Costs and consequences of a novel emergency department sepsis diagnostic test: the intellisep index. Crit Care Explor. (2023) 5:e0942. doi: 10.1097/CCE.0000000000000942 37465702 PMC10351935

[2] Rowan KM, Angus DC, Bailey M, Barnato AE, Bellomo R, Canter RR, Early, goal-directed therapy for septic shock - a patient-level meta-analysis. N Engl J Med. (2017) 376:2223–34. doi: 10.1056/NEJMoa1701380 28320242

[3] Rivers E, Nguyen B, Havstad S, Ressler J, Muzzin A, Knoblich B, Early goal-directed therapy in the treatment of severe sepsis and septic shock. N Engl J Med. (2001) 345:1368–77. doi: 10.1056/NEJMoa010307 11794169

[4] Chang DW, Tseng CH, Shapiro MF. Rehospitalizations following sepsis: common and costly. crit care med. (2015) 43:2085–93. doi: 10.1097/ccm.0000000000001159 26131597 PMC5044864

[5] Prescott HC, Langa KM, Iwashyna TJ. Readmission diagnoses after hospitalization for severe sepsis and other acute medical conditions. JAMA. (2015) 313:1055–7. doi: 10.1001/jama.2015.1410 25756444 PMC4760618

[6] Gadre SK, Shah M, Mireles-Cabodevila E, Patel B, Duggal A. Epidemiology and predictors of 30-day readmission in patients with sepsis. Chest. (2019) 155:483–90. doi: 10.1016/j.chest.2018.12.008 30846065

[7] Goodwin AJ, Ford DW. Readmissions among sepsis survivors: risk factors and prevention. Clin Pulm Med. (2018) 25:79–83. doi: 10.1097/CPM.0000000000000254 30237689 PMC6141202

[8] Goodwin AJ, Nadig NR, McElligott JT, Simpson KN, Ford DW. Where you live matters: the impact of place of residence on severe sepsis incidence and mortality. Chest. (2016) 150:829–36. doi: 10.1016/j.chest.2016.07.004 27445093 PMC5812766

[9] Lippert AM. System failure: the geographic distribution of sepsis-associated death in the usa and factors contributing to the mortality burden of black communities. J Racial Ethn Health Disparities. (2023) 10:2397–406. doi: 10.1007/s40615-022-01418-z 36171498 PMC9518946

[10] Hilton RS, Hauschildt K, Shah M, Kowalkowski M, Taylor S. The assessment of social determinants of health in postsepsis mortality and readmission: a scoping review. Crit Care Explor. (2022) 4:e0722. doi: 10.1097/CCE.0000000000000722 35928537 PMC9345631

[11] Coopersmith CM, De Backer D, Deutschman CS, Ferrer R, Lat I, Machado FR, Surviving sepsis campaign: research priorities for sepsis and septic shock. Intensive Care Med. (2018) 44:1400–26. doi: 10.1007/s00134-018-5175-z 29971592 PMC7095388

[12] Axelson DJ, Stull MJ, Coates WC. Social determinants of health: a missing link in emergency medicine training. AEM Educ Train. (2018) 2:66–8. doi: 10.1002/aet2.10056 30051070 PMC6001589

[13] Balakrishnan MP, Herndon JB, Zhang J, Payton T, Shuster J, Carden DL. The association of health literacy with preventable emergency department visits: a cross-sectional study. Acad Emerg Med. (2017) 24:1042–50. doi: 10.1111/acem.13244 28646519 PMC6535998

[14] Hwang SW, Chambers C, Chiu S, Katic M, Kiss A, Redelmeier DA, A comprehensive assessment of health care utilization among homeless adults under a system of universal health insurance. Am J Public Health. (2013) 103:S294–301. doi: 10.2105/AJPH.2013.301369 24148051 PMC3969141

[15] Alderwick H, Gottlieb LM. Meanings and misunderstandings: a social determinants of health lexicon for health care systems. Milbank Q. (2019) 97:407–19. doi: 10.1111/1468-0009.12390 31069864 PMC6554506

[16] Amrollahi F, Shashikumar SP, Meier A, Ohno-Machado L, Nemati S, Wardi G. Inclusion of social determinants of health improves sepsis readmission prediction models. J Am Med Inform Assoc. (2022) 29:1263–70. doi: 10.1093/jamia/ocac060 35511233 PMC9196687

[17] Braveman P, Gottlieb L. The social determinants of health: it’s time to consider the causes of the causes. Public Health Rep. (2014) 129:19–31. doi: 10.1177/00333549141291S206 PMC3863696 24385661

[18] Butkus R, Rapp K, Cooney TG, Engel LS. Envisioning a better US health care system for all: reducing barriers to care and addressing social determinants of health. Ann Intern Med. (2020) 172:S50–s9. doi: 10.7326/M19-2410 31958803

[19] Samuels-Kalow ME, Ciccolo GE, Lin MP, Schoenfeld EM, Camargo CA Jr. The terminology of social emergency medicine: measuring social determinants of health, social risk, and social need. J Am Coll Emerg Physicians Open. (2020) 1:852–6. doi: 10.1002/emp2.12191 33145531 PMC7593464

[20] Moreno-Juste A, Gimeno-Miguel A, Poblador-Plou B, Calderón-Larrañaga A, Cano Del Pozo M, Forjaz MJ, Multimorbidity, social determinants and intersectionality in chronic patients. Results from the EpiChron Cohort. J Glob Health. (2023) 13:04014. doi: 10.7189/13.04014 36757132 PMC9893716

[21] Galea S, Tracy M, Hoggatt KJ, Dimaggio C, Karpati A. Estimated deaths attributable to social factors in the United States. Am J Public Health. (2011) 101:1456–65. doi: 10.2105/AJPH.2010.300086 21680937 PMC3134519

[22] Chetty R, Stepner M, Abraham S, Lin S, Scuderi B, Turner N, The association between income and life expectancy in the United States, 2001–2014. Jama. (2016) 315:1750–66. doi: 10.1001/jama.2016.4226 27063997 PMC4866586

[23] Miani C, Wandschneider L, Niemann J, Batram-Zantvoort S, Razum O. Measurement of gender as a social determinant of health in epidemiology-a scoping review. PLoS ONE. (2021) 16:e0259223. doi: 10.1371/journal.pone.0259223 34731177 PMC8565751

[24] Shankar-Hari M, Saha R, Wilson J, Prescott HC, Harrison D, Rowan K, Rate and risk factors for rehospitalisation in sepsis survivors: systematic review and meta-analysis. Intensive Care Med. (2020) 46:619–36. doi: 10.1007/s00134-019-05908-3 31974919 PMC7222906

[25] Prescott HC. Preventing chronic critical illness and rehospitalization: a focus on sepsis. Crit Care Clin. (2018) 34:501–13. doi: 10.1016/j.ccc.2018.06.002 30223990

[26] Prescott HC, Langa KM, Liu V, Escobar GJ, Iwashyna TJ. Increased 1-year healthcare use in survivors of severe sepsis. Am J Respir Crit Care Med. (2014) 190:62–9. doi: 10.1164/rccm.201403-0471OC 24872085 PMC4226030

[27] Iwashyna TJ, Ely EW, Smith DM, Langa KM. Long-term cognitive impairment and functional disability among survivors of severe sepsis. JAMA. (2010) 304:1787–94. doi: 10.1001/jama.2010.1553 20978258 PMC3345288

[28] Herridge MS, Tansey CM, Matté A, Tomlinson G, Diaz-Granados N, Cooper A, Functional disability 5 years after acute respiratory distress syndrome. N Engl J Med. (2011) 364:1293–304. doi: 10.1056/NEJMoa1011802 21470008

[29] Rabiee A, Nikayin S, Hashem MD, Huang M, Dinglas VD, Bienvenu OJ, Depressive symptoms after critical illness: a systematic review and meta-analysis. Crit Care Med. (2016) 44:1744–53. doi: 10.1097/CCM.0000000000001811 27153046 PMC7418220

[30] Prescott HC, Angus DC. Enhancing recovery from sepsis: a review. JAMA. (2018) 319:62–75. doi: 10.1001/jama.2017.17687 29297082 PMC5839473

[31] Seidah NG, Prat A. The multifaceted biology of PCSK9. Endocr Rev. (2022) 43:558–82. doi: 10.1210/endrev/bnab035 35552680 PMC9113161

[32] Trinder M, Walley KR, Boyd JH, Brunham LR. Causal inference for genetically determined levels of high-density lipoprotein cholesterol and risk of infectious disease. Arterioscler Thromb Vasc Biol. (2020) 40:267–78. doi: 10.1161/ATVBAHA.119.313381 31694394 PMC6946100

[33] Barker G, Winer JR, Guirgis FW, Reddy S. HDL and persistent inflammation immunosuppression and catabolism syndrome. Curr Opin Lipidol. (2021) 32:315–22. doi: 10.1097/MOL.0000000000000782 34374677 PMC8416792

[34] Tsigalou C, Konstantinidis T, Paraschaki A, Stavropoulou E, Voidarou C, Bezirtzoglou E. Mediterranean diet as a tool to combat inflammation and chronic diseases. an overview. Biomedicines. (2020) 8:201. doi: 10.3390/biomedicines8070201 32650619 PMC7400632

[35] Yende S, Kellum JA, Talisa VB, Peck Palmer OM, Chang CH, Filbin MR, Long-term host immune response trajectories among hospitalized patients with sepsis. JAMA Netw Open. (2019) 2:e198686. doi: 10.1001/jamanetworkopen.2019.8686 31390038 PMC6686981

[36] Dankwa-Mullan I, Pérez-Stable EJ. Addressing health disparities is a place-based issue. Am J Public Health. (2016) 106:637–9. doi: 10.2105/AJPH.2016.303077 26959267 PMC4816016

[37] Cohen DA, Mason K, Bedimo A, Scribner R, Basolo V, Farley TA. Neighborhood physical conditions and health. Am J Public Health. (2003) 93:467–71. doi: 10.2105/AJPH.93.3.467 12604497 PMC1447765

[38] Johnson AE, Zhu J, Garrard W, Thoma FW, Mulukutla S, Kershaw KN, Area deprivation index and cardiac readmissions: evaluating risk-prediction in an electronic health record. J Am Heart Assoc. (2021) 10:e020466. doi: 10.1161/JAHA.120.020466 34212757 PMC8403312

[39] White K, Haas JS, Williams DR. Elucidating the role of place in health care disparities: the example of racial/ethnic residential segregation. Health Serv Res. (2012) 47:1278–99. doi: 10.1111/j.1475-6773.2012.01410.x 22515933 PMC3417310

[40] Bailey JE, Gurgol C, Pan E, Njie S, Emmett S, Gatwood J, Early patient-centered outcomes research experience with the use of telehealth to address disparities: scoping review. J Med Internet Res. (2021) 23:e28503. doi: 10.2196/28503 34878986 PMC8693194

[41] Bierman AS, Dunn JR. Swimming upstream. Access, health outcomes, and the social determinants of health. J Gen Intern Med. (2006) 21:99–100. doi: 10.1111/j.1525-1497.2005.00317.x 16423133 PMC1484628

[42] Sheehy AM, Powell WR, Kaiksow FA, Buckingham WR, Bartels CM, Birstler J, Thirty-day re-observation, chronic re-observation, and neighborhood disadvantage. Mayo Clin Proc. (2020) 95:2644–54. doi: 10.1016/j.mayocp.2020.06.059 33276837 PMC7720926

[43] Hannan EL, Wu Y, Cozzens K, Anderson B. The neighborhood atlas area deprivation index for measuring socioeconomic status: an overemphasis on home value. Health Aff. (2023) 42:702–9. doi: 10.1377/hlthaff.2022.01406 37126749

[44] Kind AJ, Jencks S, Brock J, Yu M, Bartels C, Ehlenbach W, Neighborhood socioeconomic disadvantage and 30-day rehospitalization: a retrospective cohort study. Ann Intern Med. (2014) 161:765–74. doi: 10.7326/M13-2946 25437404 PMC4251560

[45] Kind AJH, Buckingham WR. Making neighborhood-disadvantage metrics accessible - the neighborhood atlas. N Engl J Med. (2018) 378:2456–8. doi: 10.1056/NEJMp1802313 29949490 PMC6051533

[46] Hilmers A, Hilmers DC, Dave J. Neighborhood disparities in access to healthy foods and their effects on environmental justice. Am J Public Health. (2012) 102:1644–54. doi: 10.2105/AJPH.2012.300865 22813465 PMC3482049

[47] McCullough ML, Chantaprasopsuk S, Islami F, Rees-Punia E, Um CY, Wang Y, Association of socioeconomic and geographic factors with diet quality in US adults. JAMA Netw Open. (2022) 5:e2216406. doi: 10.1001/jamanetworkopen.2022.16406 35679041 PMC9185183

[48] Diez Roux AV, Mair C. Neighborhoods and health. Ann N Y Acad Sci. (2010) 1186:125–45. doi: 10.1111/j.1749-6632.2009.05333.x 20201871

[49] Walker RE, Keane CR, Burke JG. Disparities and access to healthy food in the United States: a review of food deserts literature. Health Place. (2010) 16:876–84. doi: 10.1016/j.healthplace.2010.04.013 20462784

[50] Ver Ploeg M, Breneman V, Farrigan T, Hamrick K, Hopkins D, Kaufman P, Access to Affordable and Nutritious Food: Measuring and Understanding Food Deserts and their Consequences: Report to Congress. (2009). Available online at: https://www.ers.usda.gov/publications/pub-details/?pubid=42729

[51] Kelli HM, Hammadah M, Ahmed H, Ko YA, Topel M, Samman-Tahhan A, Association between living in food deserts and cardiovascular risk. Circ Cardiovasc Qual Outcomes. (2017) 10:e003532. doi: 10.1161/CIRCOUTCOMES.116.003532 28904075 PMC5810926

[52] Testa A, Jackson DB, Semenza DC, Vaughn MG. Food deserts and cardiovascular health among young adults. Public Health Nutr. (2021) 24:117–24. doi: 10.1017/S1368980020001536 32641177 PMC10195490

[53] Kelli HM, Kim JH, Tahhan AS, Liu C, Ko YA, Hammadah M, Living in food deserts and adverse cardiovascular outcomes in patients with cardiovascular disease. J Am Heart Assoc. (2019) 8:e010694. doi: 10.1161/JAHA.118.010694 30741595 PMC6405658

[54] Warburton DE, Nicol CW, Bredin SS. Health benefits of physical activity: the evidence. Cmaj. (2006) 174:801–9. doi: 10.1503/cmaj.051351 16534088 PMC1402378

[55] Kim M-H, Sung J-H, Jin M-N, Jang E, Yu HT, Kim T-H, Impact of physical activity on all-cause mortality according to specific cardiovascular disease. Front Cardiovasc Med. (2022) 9:811058. doi: 10.3389/fcvm.2022.811058 35187126 PMC8855984

[56] Löllgen H, Böckenhoff A, Knapp G. Physical activity and all-cause mortality: an updated meta-analysis with different intensity categories. Int J Sports Med. (2009) 30:213–24. doi: 10.1055/s-0028-1128150 19199202

[57] Zhao M, Veeranki SP, Magnussen CG, Xi B. Recommended physical activity and all cause and cause specific mortality in US adults: prospective cohort study. BMJ. (2020) 370:m2031. doi: 10.1136/bmj.m2031 32611588 PMC7328465

[58] de Koning IA, van Bakel BMA, Rotbi H, Van Geuns RM, Cramer GE, Pop GAM, Association between engagement in exercise training and peak cardiac biomarker concentrations following ST-elevation myocardial infarction. BMJ Open Sport Exerc Med. (2023) 9:e001488. doi: 10.1136/bmjsem-2022-001488 PMC10106052 37073175

[59] Shi J, Liang Z, Zhang X, Ren S, Cheng Y, Liu Y, Association of physical activity and dietary inflammatory index with overweight/obesity in US adults: NHANES 2007–2018. Environ Health Prev Med. (2023) 28:40. doi: 10.1265/ehpm.23-00016 37380500 PMC10331001

[60] O’Donoghue G, Kennedy A, Puggina A, Aleksovska K, Buck C, Burns C, Socio-economic determinants of physical activity across the life course: a “determinants of diet and physical activity” (DEDIPAC) umbrella literature review. PLoS ONE. (2018) 13:e0190737. doi: 10.1371/journal.pone.0190737 29351286 PMC5774703

[61] Corl K, Levy M, Phillips G, Terry K, Friedrich M, Trivedi AN. Racial and ethnic disparities in care following the new york state sepsis initiative. Health Aff. (2019) 38:1119–26. doi: 10.1377/hlthaff.2018.05381 PMC6814952 31260359

[62] Taylor SP, Karvetski CH, Templin MA, Taylor BT. Hospital differences drive antibiotic delays for black patients compared with white patients with suspected septic shock. Crit Care Med. (2018) 46:e126–e31. doi: 10.1097/CCM.0000000000002829 29116997

[63] Honda TJ, Kazemiparkouhi F, Henry TD, Suh HH. Long-term PM(25) exposure and sepsis mortality in a US medicare cohort. BMC Public Health. (2022) 22:1214. doi: 10.1186/s12889-022-13628-5 35717154 PMC9206363

[64] Galiatsatos P, Follin A, Alghanim F, Sherry M, Sylvester C, Daniel Y, The association between neighborhood socioeconomic disadvantage and readmissions for patients hospitalized with sepsis. Crit Care Med. (2020) 48:808–14. doi: 10.1097/CCM.0000000000004307 32271185 PMC7391606

[65] Lusk JB, Blass B, Mahoney H, Hoffman MN, Clark AG, Bae J, Neighborhood socioeconomic deprivation, healthcare access, and 30-day mortality and readmission after sepsis or critical illness: findings from a nationwide study. Crit Care. (2023) 27:287. doi: 10.1186/s13054-023-04565-9 37454127 PMC10349422

[66] Iwashyna TJ, Burke JF, Sussman JB, Prescott HC, Hayward RA, Angus DC. Implications of heterogeneity of treatment effect for reporting and analysis of randomized trials in critical care. Am J Respir Crit Care Med. (2015) 192:1045–51. doi: 10.1164/rccm.201411-2125CP 26177009 PMC4642199

[67] Mayr FB, Yende S, Linde-Zwirble WT, Peck-Palmer OM, Barnato AE, Weissfeld LA, Infection rate and acute organ dysfunction risk as explanations for racial differences in severe sepsis. JAMA. (2010) 303:2495–503. doi: 10.1001/jama.2010.851 20571016 PMC3910506

[68] Rubens M, Saxena A, Ramamoorthy V, Das S, Khera R, Hong J, Increasing sepsis rates in the united states: results from national inpatient sample, 2005 to 2014. J Intensive Care Med. (2020) 35:858–68. doi: 10.1177/0885066618794136 30175649

[69] Tyler PD, Stone DJ, Geisler BP, McLennan S, Celi LA, Rush B. Racial and geographic disparities in interhospital ICU transfers. Crit Care Med. (2018) 46:e76–80. doi: 10.1097/CCM.0000000000002776 29068859 PMC5743219

[70] Barnato AE, Alexander SL, Linde-Zwirble WT, Angus DC. Racial variation in the incidence, care, and outcomes of severe sepsis: analysis of population, patient, and hospital characteristics. Am J Respir Crit Care Med. (2008) 177:279–84. doi: 10.1164/rccm.200703-480OC 17975201 PMC2720103

[71] Moore JX, Donnelly JP, Griffin R, Howard G, Safford MM, Wang HE. Defining sepsis mortality clusters in the United States. Crit Care Med. (2016) 44:1380–7. doi: 10.1097/CCM.0000000000001665 27105174 PMC4911271

[72] DiMeglio M, Dubensky J, Schadt S, Potdar R, Laudanski K. Factors underlying racial disparities in sepsis management. Healthcare. (2018) 6:133. doi: 10.3390/healthcare6040133 30463180 PMC6315577

[73] Kumar G, Taneja A, Majumdar T, Jacobs ER, Whittle J, Nanchal R. The association of lacking insurance with outcomes of severe sepsis: retrospective analysis of an administrative database*. Crit Care Med. (2014) 42:583–91. doi: 10.1097/01.ccm.0000435667.15070.9c 24152590 PMC4990450

[74] Sandoval E, Chang DW. Association between race and case fatality rate in hospitalizations for sepsis. J Racial Ethn Health Disparities. (2016) 3:625–34. doi: 10.1007/s40615-015-0181-0 27294755

[75] Nathanson BH, Higgins TL, Stefan M, Lagu T, Lindenauer PK, Steingrub JS. An analysis of homeless patients in the United States requiring ICU admission. J Crit Care. (2019) 49:118–23. doi: 10.1016/j.jcrc.2018.10.026 30419544

[76] Lakbar I, Einav S, Lalevée N, Martin-Loeches I, Pastene B, Leone M. Interactions between gender and sepsis-implications for the future. Microorganisms. (2023) 11:746. doi: 10.3390/microorganisms11030746 36985319 PMC10058943

[77] Lakbar I, Munoz M, Pauly V, Orleans V, Fabre C, Fond G, Septic shock: incidence, mortality and hospital readmission rates in French intensive care units from 2014 to 2018. Anaesth Crit Care Pain Med. (2022) 41:101082. doi: 10.1016/j.accpm.2022.101082 35472583

[78] Goodwin AJ, Rice DA, Simpson KN, Ford DW. Frequency, cost, and risk factors of readmissions among severe sepsis survivors. Crit Care Med. (2015) 43:738–46. doi: 10.1097/CCM.0000000000000859 25746745 PMC4479267

[79] Zhang D, Chen W, Cheng C, Huang H, Li X, Qin P, Air pollution exposure and heart failure: a systematic review and meta-analysis. Sci Total Environ. (2023) 872:162191. doi: 10.1016/j.scitotenv.2023.162191 36781139

[80] Zilbermint M, Hannah-Shmouni F, Stratakis CA. Genetics of Hypertension in African Americans and Others of African Descent. Int J Mol Sci. (2019) 20:1081. doi: 10.3390/ijms20051081 30832344 PMC6429313

[81] Kirby JB, Kaneda T. Neighborhood socioeconomic disadvantage and access to health care. J Health Soc Behav. (2005) 46:15–31. doi: 10.1177/002214650504600103 15869118

[82] House T, Keeling MJ. Household structure and infectious disease transmission. Epidemiol Infect. (2009) 137:654–61. doi: 10.1017/S0950268808001416 18840319 PMC2829934

[83] Noppert GA, Wilson ML, Clarke P, Ye W, Davidson P, Yang Z. Race and nativity are major determinants of tuberculosis in the U.S.: evidence of health disparities in tuberculosis incidence in Michigan, 2004–2012. BMC Public Health. (2017) 17:538. doi: 10.1186/s12889-017-4461-y 28578689 PMC5457589

[84] Shi T, Balsells E, Wastnedge E, Singleton R, Rasmussen ZA, Zar HJ, Risk factors for respiratory syncytial virus associated with acute lower respiratory infection in children under five years: systematic review and meta-analysis. J Glob Health. (2015) 5:020416. doi: 10.7189/jogh.05.020416 26682048 PMC4676580

[85] Link BG, Phelan J. Social conditions as fundamental causes of disease. J Health Soc Behav. (1995) Spec No:80–94. doi: 10.2307/2626958 7560851

[86] Noppert GA, Hegde ST, Kubale JT. Exposure, susceptibility, and recovery: a framework for examining the intersection of the social and physical environments and infectious disease risk. Am J Epidemiol. (2023) 192:475–82. doi: 10.1093/aje/kwac186 36255177 PMC10372867

[87] Cook LA, Sachs J, Weiskopf NG. The quality of social determinants data in the electronic health record: a systematic review. J Am Med Inform Assoc. (2021) 29:187–96. doi: 10.1093/jamia/ocab199 34664641 PMC8714289

[88] Lett E, Asabor E, Beltrán S, Cannon AM, Arah OA. Conceptualizing, contextualizing, and operationalizing race in quantitative health sciences research. Ann Fam Med. (2022) 20:157–63. doi: 10.1370/afm.2792 35045967 PMC8959750

[89] Truong HP, Luke AA, Hammond G, Wadhera RK, Reidhead M, Joynt Maddox KE. Utilization of social determinants of health ICD-10 Z-codes among hospitalized patients in the United States, 2016–2017. Med Care. (2020) 58:1037–43. doi: 10.1097/MLR.0000000000001418 32925453 PMC7666017

[90] Arday SL, Arday DR, Monroe S, Zhang J. HCFA’s racial and ethnic data: current accuracy and recent improvements. Health Care Financ Rev. (2000) 21:107–16. 11481739 PMC4194641

[91] Jarrín OF, Nyandege AN, Grafova IB, Dong X, Lin H. Validity of race and ethnicity codes in medicare administrative data compared with gold-standard self-reported race collected during routine home health care visits. Med Care. (2020) 58:e1–8. doi: 10.1097/MLR.0000000000001216 31688554 PMC6904433

[92] Huang KE, Lipsitch M, Shaman J, Goldstein E. The US 2009 A(H1N1) influenza epidemic: quantifying the impact of school openings on the reproductive number. Epidemiology. (2014) 25:203–6. doi: 10.1097/EDE.0000000000000055 24434751 PMC3960948

[93] Lowen AC, Steel J. Roles of humidity and temperature in shaping influenza seasonality. J Virol. (2014) 88:7692–5. doi: 10.1128/JVI.03544-13 24789791 PMC4097773

[94] Goldstein E, Pitzer VE, O’Hagan JJ, Lipsitch M. Temporally varying relative risks for infectious diseases: implications for infectious disease control. Epidemiology. (2017) 28:136–44. doi: 10.1097/EDE.0000000000000571 27748685 PMC5131868

[95] Allred EN, Dammann O, Kuban K, Leviton A, Pagano M. Neonatal risk factors for cerebral palsy in very preterm babies - time oriented analyses of risk are useful. Br Med J. (1997) 314:1624. doi: 10.1136/bmj.314.7094.1624 PMC2126798 9186191

[96] Ohno-Machado L Modeling medical prognosis: survival analysis techniques. J Biomed Inform. (2001) 34:428–39. doi: 10.1006/jbin.2002.1038 12198763

[97] Leviton A, Kuban K, O’Shea TM, Paneth N, Fichorova R, Allred EN, The Relationship between early concentrations of 25 blood proteins and cerebral white matter injury in preterm newborns: the ELGAN study. J Pediatr. (2011) 158:897–903.e5. doi: 10.1016/j.jpeds.2010.11.059 21238986

[98] Kowalkowski MA, Rios A, McSweeney J, Murphy S, McWilliams A, Chou SH, Effect of a transitional care intervention on rehospitalization and mortality after sepsis: a 12-month follow-up of a randomized clinical trial. Am J Respir Crit Care Med. (2022) 206:783–6. doi: 10.1164/rccm.202203-0590LE 35608544

[99] Taylor SP, Murphy S, Rios A, McWilliams A, McCurdy L, Chou SH, Effect of a multicomponent sepsis transition and recovery program on mortality and readmissions after sepsis: the improving morbidity during post-acute care transitions for sepsis randomized clinical trial. Crit Care Med. (2022) 50:469–79. doi: 10.1097/CCM.0000000000005300 34534130 PMC10229099

[100] Singer M, Bulled N, Ostrach B, Mendenhall E. Syndemics and the biosocial conception of health. Lancet. (2017) 389:941–50. doi: 10.1016/S0140-6736(17)30003-X 28271845

[101] Rudd KE, Mair CF, Angus DC. Applying syndemic theory to acute illness. JAMA. (2022) 327:33–4. doi: 10.1001/jama.2021.22583 34919123 PMC8769249

[102] Kellermann AL. Emergency medicine and public health: stopping emergencies before the 9-1-1 call. Acad Emerg Med. (2009) 16:1060–4. doi: 10.1111/j.1553-2712.2009.00567.x 20053223

[103] Hsieh YH, Jung JJ, Shahan JB, Moring-Parris D, Kelen GD, Rothman RE. Emergency medicine resident attitudes and perceptions of HIV testing before and after a focused training program and testing implementation. Acad Emerg Med. (2009) 16:1165–73. doi: 10.1111/j.1553-2712.2009.00507.x 20053237

[104] Macy ML, Clark SJ, Sasson C, Meurer WJ, Freed GL. Emergency physician perspectives on child passenger safety: a national survey of attitudes and practices. Acad Pediatr. (2012) 12:131–7. doi: 10.1016/j.acap.2011.10.002 22104388

